# Photodynamic therapy using 5-aminolaevulinic acid for experimental pancreatic cancer--prolonged animal survival.

**DOI:** 10.1038/bjc.1994.288

**Published:** 1994-08

**Authors:** J. Regula, B. Ravi, J. Bedwell, A. J. MacRobert, S. G. Bown

**Affiliations:** Department of Surgery, University College London Medical School, Rayne Institute, UK.

## Abstract

**Images:**


Br. J. Cancer (1994), 70, 248-254 © Macmillan Press Ltd., 1994

### Photodynamic therapy using 5-aminolaevulinic acid for experimental pancreatic cancer – prolonged animal survival

J. Regula[1,2], B. Ravi[1,3], J. Bedwell[1], A.J. MacRobert[1] & S.G. Bown[1]

[1]*National Medical Laser Centre, Department of Surgery, University College London Medical School, The Rayne Institute, 5 University Street, London WC1E 6JJ, UK;* [2]*Department of Gastroenterology, Medical Centre for Postgraduate Education, Goszczynskiego Street 1, 02-616 Warsaw, Poland;* [3]*Department of Surgery, The Medical College, Rohtak-124001, India.*

**Summary** Experimental studies have been carried out using 5-aminolaevulinic acid (ALA) to induce transient porphyrin photosensitisation for photodynamic therapy (PDT) in a pancreatic cancer model in Syrian golden hamsters. ALA was given either intravenously or orally (in bolus or fractionated doses) with the laser light delivered by means of a bare fibre touching the tissue surface or external irradiation using a light-integrating cylindrical applicator. Animals were killed 1-24 h after ALA administration for pharmacokinetic studies and 3-7 days after light exposure to study PDT-induced necrosis. A separate survival study was also performed after a fractionated oral dose of ALA and external irradiation. Protoporphyrin IX sensitisation in the tumour tissue as measured by quantitative fluorescence microscopy was highest after intravenous administration of 200 mg kg$^{-1}$ ALA and then in decreasing order after oral fractionated and oral bolus doses (both 400 mg kg$^{-1}$). Laser light application at 630 nm to give 12-50 J from the bare fibre or 50 J cm$^{-2}$ using surface illumination with the cylindrical applicator resulted in tumour necrosis up to 8 mm in depth. In larger tumours a rim of viable tumour was observed on the side opposite to illumination. In a randomised study, survival of treated animals was significantly longer than in the untreated control group (log-rank test, $P < 0.02$), although all animals died of recurrent tumour. This technique shows promise in the treatment of small volumes of tumour in the pancreas.

Pancreatic cancer has one of the poorest prognoses of any cancer. Most patients die within 6 months of diagnosis. The 1 and 5 year survival rates are about 10% and 1-2% respectively. It is one of the most common cancers of the gastrointestinal tract, and its incidence is increasing. Radical surgery is rarely possible, and radiotherapy and chemotherapy are of no proven benefit. Photodynamic therapy (PDT) is a new and promising approach to therapy for tumours. It is a non-thermal technique for producing localised tissue necrosis with light-activated photosensitising compounds. Systemic administration of the photosensitiser is followed by exposure to laser light at a wavelength matched to an absorption peak of the sensitiser, resulting in the generation of cytotoxic singlet oxygen. Ideally, the photosensitiser should be selectively retained in the tumour and not in the surrounding normal tissue. This would ensure safe destruction of the tumour within normal areas. However, even if there is some selectivity in the tumour uptake of the photosensitiser, truly selective destruction is difficult to obtain and therefore some degree of normal tissue damage has to be accepted provided safe healing can occur (Bown, 1990). Haematoporphyrin derivative (HpD) and its purer commercial variants (e.g. Photofrin) are the most commonly used photosensitisers in clinical practice. They are both rather poorly defined mixtures of porphyrins and have the clinically important disadvantage of causing prolonged skin photosensitivity lasting for about 4-6 weeks (Dougherty *et al.*, 1990).

Pancreatic cancer has never been treated by PDT in humans, but experimental studies on pancreatic tumour models in hamsters or rats have been carried out (Mang & Wieman, 1987; Schroder *et al.*, 1988; Chatlani *et al.*, 1992). The common conclusions drawn from those studies are encouraging. Necrosis of the pancreatic cancer was achieved easily and, more importantly, normal pancreatic tissue surrounding the tumour showed resistance to PDT damage (Mang & Wieman, 1987; Nuutinen *et al.*, 1991; Chatlani *et al.*, 1992). Photofrin or aluminium sulphonated phthalocyanine (AlSPc) was used as the photosensitiser in these studies. AlSPc has better characteristics than Photofrin, causing much less severe and less prolonged skin photosensitivity (Tralau *et al.*, 1989), but its clinical use is not yet approved. Another particularly promising approach is the use of 5-aminolaevulinic acid (ALA), and recently this means of photosensitisation has been studied intensively. ALA is a naturally occurring precursor in the biosynthetic chain for haem production. The last step, which is rate limiting, involves conversion of the photosensitising species protoporphyrin IX (PPIX) to haem. Upon exogenous administration ALA may be quickly metabolised to PPIX, which then accumulates in the tissues with consequent endogenous photosensitisation that can be exploited for PDT (Malik & Lugaci, 1987; Divaris *et al.*, 1990). Favourable results have been obtained in the treatment of animal tumour models (Bedwell *et al.*, 1992; Peng *et al.*, 1992) and also after topical application of ALA for 300 cutaneous basal cell carcinoma lesions in Canada (Kennedy & Pottier, 1992) and in oral cancer patients following systemic ALA administration (Grant *et al.*, 1993). The main advantages of using ALA as a photosensitising agent lie in the rapid elimination of ALA from the body, short-lived photosensitisation lasting not longer than 24 h and the possibility of oral administration, which is more acceptable and easier than any parenteral route (Berlin *et al.*, 1956; Mustajoki *et al.*, 1992; Grant *et al.*, 1993; Loh *et al.*, 1993*a*). ALA-induced PPIX sensitisation has not been studied previously in experimental pancreatic cancer. Additionally, none of the aforementioned studies with Photofrin or AlSPc sensitisation in pancreatic tumour models explored whether PDT could improve the survival of treated animals.

The aim of this study was to investigate the possibility of using ALA-induced PPIX for PDT for a transplanted pancreatic cancer in the Syrian golden hamster. We studied PPIX sensitisation kinetics and laser irradiation effects in the pancreatic tumour and normal pancreas after intravenous and oral administration of ALA. Additionally, in a separate study, the survival time after PDT was compared with a control, non-treated group of tumour-bearing animals.

### Materials and methods

### *Tumour model*

Female Syrian golden hamsters weighing 80-120 g were used in all experiments. Laparotomies were performed under int-

Correspondence: S.G. Bown
Received 14 December 1993; and in revised form 31 March 1994.

raperitoneal injection of xylazine $10\,mg\,kg^{-1}$ and ketamine $200\,mg\,kg^{-1}$, which provided good general anaesthesia. A pancreatic tumour was established at laparotomy by intrapancreatic (into the gastric lobe) injection of $10^7$ cells from the pancreatic cancer line (PC-1), which was obtained from The Eppley Institute for Research in Cancer and Allied Diseases, Univerity of Nebraska Medical Centre, Omaha, USA. The PC-1 line has been described in detail elsewhere (Egami *et al.*, 1989) and was derived from a hamster pancreatic cancer induced by *N*-nitrosobis(2-oxopropyl)amine (BOP). This model retains the histological, biological and antigenic properties of the primary ductal carcinoma and is very similar to human disease (Takiyama *et al.*, 1990). The cell culture medium consisted of RPMI-1640 supplemented with penicillin G (5,000 IU $ml^{-1}$), streptomycin (5,000 $\mu g\,ml^{-1}$) (all from Flow Laboratories, Irvine, UK), 10% fetal calf serum (Advanced Protein products, UK) and L-glutamine 200 mM solution (Sigma, UK). For intrapancreatic injection, cultured cells were detached from the bottom of plastic culture flasks by 0.05% trypsin and 0.02% EDTA solution (Boehringer Mannheim, Germany) and were suspended in 0.2–0.3 ml of normal saline. Three weeks after the injection, pancreatic tumour could be detected in the gastric lobe of the pancreas at laparotomy in about 80% of animals (mean diameter of the tumour was $1.4 \pm 0.6$ cm). Spontaneous necrosis in the centre of the largest tumours was confined to a region $<4$ mm in diameter.

### *Fluorescence microscopy studies*

5-Aminolaevulinic acid (ALA) was obtained from Sigma (UK) with a purity of 98%. Three different systemic routes of ALA administration were used for hamster photosensitisation: (a) intravenous (i.v.), in which ALA was dissolved in phosphate-buffered saline (PBS) at a concentration of $40\,mg\,ml^{-1}$ and injected into the inferior vena cava at laparotomy (total dose $200\,mg\,kg^{-1}$); (b) oral bolus (o.b.) in which ALA, dissolved in 1 ml of PBS, was injected into the stomach by a long, bulb-tip feeding needle (dose $400\,mg\,kg^{-1}$); and (c) oral fractionated (o.f.), in which the ALA was first dissolved in 5 ml of saline and then injected in 1 ml doses at 1 h intervals into the stomach by the feeding needle ($5 \times 80\,mg\,kg^{-1}$; total dose $400\,mg\,kg^{-1}$). A higher total dose of ALA for enteral dosages (o.b. and o.f. $400\,mg\,kg^{-1}$) than for the i.v. route ($200\,mg\,kg^{-1}$) was chosen because of likely first-pass liver metabolism after oral administration, following the results of Loh *et al.* (1993*a*), who showed that giving an oral ALA dose twice that of the intravenous dose resulted in similar fluorescence levels in the rat stomach, bladder and colon. At 1, 2, 3, 4, 5, 6, 7, 8 or 24 h following either i.v. or o.b. administration of ALA animals were killed and tumour and normal pancreas removed and immediately frozen in a bath of isopentane (BDH, UK) precooled in liquid nitrogen and then stored in liquid nitrogen. For o.f. dosing the same time points were used but refer to the time elapsed *after the first* fractioned dose, and the first animals were not killed for 4 h, i.e. just after the last dose was given. Fluorescence microscopy studies as described in detail elsewhere (Bedwell *et al.*, 1992) were performed on 10-μm-thick frozen sections cut with a Cryocut E microtome (Reichert). An inverted microscope (IMT-2, Olympus) with epifluorescence and phase-contrast attachments and with a slow-scan, cooled, charged-coupled device (CCD) camera (Wright Instruments) was used to obtain fluorescence images of the selected area of the section. A $10\times$ objective was used throughout to give images of $880 \times 550\,\mu m$ dimensions. Fluorescence was excited using an 8 mW helium neon laser (632.8 nm wavelength) and detected in the range 660–710 nm using a combination of bandpass (Omega Optical) and longpass (Schott RG655) filters. The fluorescence signal was processed by an IBM computer generating false colour-coded images. Digital quantification of the fluorescence intensity from the areas of interest was measured in arbitrary units of counts per pixel. This combination of laser-induced fluorescence and the slow-scan CCD imaging technique has proven to be sufficiently highly sensitive and reproducible for microscopic pharmacokinetic studies (Bedwell *et al.*, 1992). Previous experiments correlating emission spectroscopy of *ex vivo* tissue specimens and high-performance liquid chromatographic (HPLC) analysis of chemically extracted prophyrins from rat tissues (Loh *et al.*, 1993*a,b*) have shown that PPIX is the predominant ($>95\%$) porphyrin present after ALA administration. After fluorescence images were recorded, the same sections were fixed in formalin and stained with haematoxylin and eosin for comparative light microscopy analysis, which allowed precise identification of the fluorescing structures. Control, unsensitised tissues were also examined to quantify the autofluorescence intensity levels.

### *Photodynamic therapy–short – term study*

To assess whether ALA administration followed by laser light illumination can induce necrosis and, if so, what is the extent of this necrosis in our pancreatic tumour model, we initially performed a short-term study. Pancreatic tumours in 13 animals were treated with the laser light at laparotomy 21 ($\pm$ 1) days after implantation of PC-1 cells, when the mean tumour diameter was about 1.4 cm. Since the highest fluorescence levels in the tumours were seen 4 h after i.v. administration and 5 h after the fractionated dosage (o.f.), these were the time points chosen for the laser irradiation when resultant tumour damage was likely to be maximal, although not corresponding to the best ratio between tumour and normal pancreas. Oral bolus dosage (o.b.) was not used since on the basis of the fluorescence microscopy studies o.b. ALA administration gave the weakest peak PPIX fluorescence. The light source was a copper vapour pumped-dye laser (Oxford Lasers) set to deliver red light at 630 nm. There were two main methods of light delivery. In method 1, the laser light was coupled to a 200 μm bare fibre which was just touching the surface of the tumour to deliver the chosen energy of 50 J (50 mW for 1,000 s); in method 2 we used a 400 μm microlens fibre mounted inside a light-integrating cylinder with a reflective inner surface (2.5 cm long, fibre position 2 cm above the area to be irradiated), which enabled more selective tumour illumination with a surface irradiance of $50\,J\,cm^{-2}$ ($100\,mW\,cm^{-2}$ for 500 s). Animals were killed 3, 5, 6 or 7 days later and, at autopsy, damage to the tumour and surrounding normal tissues was assessed macroscopically and relevant samples collected for histological analysis.

### *Survival study*

Twenty hamsters weighing 110 g ($\pm$ 10 g) were injected with PC-1 cells as described above with the intention of performing a control laparotomy for tumour assessment 3 weeks later. One animal had to be killed the day after PC-1 injection because of an incisional hernia. Of the remaining 19 animals, control laparotomy performed 20–22 days after tumour implantation revealed that four had no tumour and that two had developed liver metastases and so these six animals were excluded from the survival study. All animals, including those from the control group, received the first oral fractionated dose of ALA 5 h before laparotomy and randomisation. Only animals with suitable tumours were randomised. Hence, 13 tumour-bearing hamsters were randomised to either the treatment or control group according to our code, allowing for about twice as many animals in the active treatment group. As a result of randomisation, nine hamsters with a mean ($\pm$ standard deviation) tumour diameter of 1.4 ($\pm$ 0.6 cm) underwent treatment with irradiation method 2 described above, and four hamsters with a mean tumour diameter of $1.3 \pm 0.5$ cm formed the control, untreated group. The tumour diameters in the two groups did not differ statistically (Mann–Whitney *U*-test, $P = 0.81$). Method 2 was chosen for light exposure because we thought that shielding of the normal tissues was desirable in the long-term survival study. The oral fractionated dosage was chosen because the intravenous method required two laparotomies on the treatment day and the oral bolus dose gave weaker fluorescence in the tumour than the fractionated one. After laparotomy and wound repair, animals were allowed to recover and then observed every 2 days. Survival time was defined as the time from the control laparotomy (with or without laser treatment) to death. All animals underwent necropsy examination. Six hamsters from the treated group and two from the control group were killed when they became distressed because of carcinomatosis (respiratory failure with ascites). Care was taken to apply the same criteria of distress in all animals. These studies were carried out in accordance with procedures approved by the Home Office.

### *Statistical analysis*

Fluorescence intensity at a given time point was expressed as a mean of measurements obtained from 2-4 animals after subtracting autofluorescence intensity obtained from the same tissues in non-sensitised animals. Each animal had fluorescence measured in the tumour and normal pancreas at two sites in at least two frozen sections. For survival analysis Kaplan-Meier curves were created and survival probabilities compared with the log-rank test. A difference was considered to be significant at a $P$-value less than 0.05.

### Results

### *Fluorescence microscopy*

Low fluorescence intensity levels not exceeding 20 counts per pixel at any time point were detected in the normal pancreas regardless of the route of ALA administration (Figure 1). The highest values were obtained in the pancreatic tumour after intravenous administration of ALA with a peak of 97 counts per pixel at 4 h. The peak time point was similar for an oral bolus dose (at 4 h), but the fluorescence intensity was only half the level of that after i.v., despite the higher dose given. Oral fractionated dosage gave fluorescence levels lower than after i.v. but higher than after o.b. administration. Peak values were detected at 5 h after the first dose, i.e. 1 h after the last fractionated dose. An example of a fluorescence image from tumour and adjacent normal pancreas at the 5 h time-point is shown in Figure 2, which demonstrates the marked tumour selectivity in fluorescence levels. Table I gives the values and times of maximum tumour fluorescence for each administration route together with the corresponding fluorescence ratio between tumour and normal pancreas.

### *Photodynamic therapy-short - term results*

The earliest results were obtained 3 days and the latest 7 days after treatment. In all treated tumours ($n = 13$) necrosis was evident, and the largest necrotic area had a diameter of 0.8 cm. Progression of the necrotic changes could be seen on serial slides obtained on days 3, 5, 6 and 7. On day 3 the 'skeleton of the tumour' (i.e. damaged but recognisable histological structure of the tumour) could still be seen, but on day 7 mostly just amorphous necrotic material was present. Smaller tumours showed complete necrosis of the entire lesion, but larger ones usually had a rim of viable tumour on the side opposite the irradiated surface (Figure 3). The distance from the irradiated surface of the tumour to the viable tumour rim was up to 0.8 cm. The border between necrosed and viable tumour was uneven and had a tendency to follow

**Figure 1** Microscopic fluorescence intensity in pancreatic tumour **a**, and normal pancreas **b**, after i.v. (□, 200 mg $kg^{-1}$), o.f. (◆, 400 mg $kg^{-1}$) and o.b. (■, 400 mg $kg^{-1}$) ALA as a function of time after administration. At 24 h after ALA, fluorescence intensities returned to background values in all tissues studied (not shown on graphs).

**Figure 2** **a**, Fluoresence images of pancreatic tumour and normal pancreas 5 h after o.f. (400 mg $kg^{-1}$) ALA. The highest fluorescence is represented by white colour, the lowest by black, according to the scale bar (from 35 to 128 counts per pixel) in the upper part of the picture. **b**, The same section stained with haematoxylin and eosin (T, tumour; N, normal pancreas). Scale bar represents 80 μm.

**Table I** Maximum tumour microfluorimetric fluorescence levels and selectivity ratios for tumour versus pancreas with intravenous or oral administration

| | *Intravenous (200 mg kg⁻¹)* | *Oral bolus (400 mg kg⁻¹)* | *Oral fractionated (5 × 80 mg kg⁻¹)* |
|---|---|---|---|
| Time of maximum tumour fluorescence (h) | 4 | 4 | 5[a] |
| Tumour/counts (± s.d.) | 97 (11) | 42 (15) | 65 (15) |
| Pancreas/counts (± s.d.) | 18 (4) | 16 (5) | 10 (3) |
| Fluorescence ratio (tumour/pancreas) | 5:1 | 3:1 | 6:1 |

[a]For fractionated oral administration this time represents 5 h after the first fractionated dose.

**Figure 3** Histological section of pancreatic tumour treated with 50 J cm$^{-2}$ 5 h after oral fractionated 400 mg kg$^{-1}$ ALA showing necrotic tumour area (NT) and the rim of viable tumour (VT) at the opposite side to illuminated surface. Scale bar represents 1 mm.

**Figure 4** Histological section of pancreatic tumour treated with 50 J from bare fibre 4 h after i.v. 200 mg kg$^{-1}$ ALA showing that the borders between necrotic tumour (NT) and viable tumour (VT) follow the lobular structure of the tumour. Scale bar represents 1 mm.

the lobular structure of the tumour delineated by thin connective tissue 'strips' (Figure 4), which may be related to the vascular supply to the different parts of the tumour or to the protective role of connective tissue strips. The extent of the tumour necrosis did not depend on the method of irradiation and was similar for methods 1 and 2.

Normal pancreatic tissue present at the borders of the tumour and surrounding it in most of the sections was not damaged. However, usually small areas of pancreatic oedema or even necrosis could be detected, but only in places where direct irradiation took place. Control animals with untreated tumour killed 28 days after tumour implantation (similar time of observation as the longest observation period in the treated group, namely 7 days after treatment) exhibited only small areas of spontaneous tumour necrosis located in the middle part of the tumour, which was in marked contrast to the location of the PDT necrosis, which extended from the borders of the tumour.

### *Survival study*

The mean survival time of control untreated animals was 42 days (range 29–51). Animals treated with PDT after an oral fractionated dose of ALA (400 mg kg$^{-1}$) irradiated at 5 h with the cylindrical applicator (50 J cm$^{-2}$) survived significantly longer according to the Kaplan–Meier analysis of survival curves (log-rank test; $\chi^2 = 5.86$, $P < 0.02$) (Figure 5). In all controls and six of the treated animals death was caused by abdominal carcinomatosis with ascites. Two treated animals died as a result of duodenal infiltration and malnutrition: no distant metastases could be detected on autopsy. The remaining hamster from the treated group lived up to 116 days in apparently good health without symptoms (to become a censored observation in the Kaplan–Meier analysis) and was killed. On autopsy there was a 2 cm pancreatic tumour infiltrating liver and duodenum and slight ascites. No damage to the surrounding normal tissues that could be related to laser treatment was noticed in any of the animals from the treated group. In particular, there was no evidence that laser treatment had caused any stenosis or perforation of the stomach, duodenum or biliary tree.

### Discussion

There are only three published studies addressing PDT treatment of experimental pancreatic cancer. However, the methods used to induce the tumour in these studies were different from our model. In the first study two models were employed (Mang & Wieman, 1987). One was a rat pancreatic carcinoma originally induced by azaserine and subsequently maintained by serial transplantation into the same strains of rat subcutaneously and intraperitoneally, which resulted in growth of acinar cell-type carcinomas. In the second model a pancreatic ductal-type adenocarcinoma was induced in hamsters by *N*-nitrosobis(2-oxopropyl)amine (BOP) injections and serial subcutaneous and intrapancreatic transplantations. In the two other studies (Schroeder *et al.*, 1988; Chatlani *et al.*, 1992) Syrian golden hamsters were injected subcutaneously with BOP to induce pancreatic ductal carcinomas. Our method was described by Egami *et al.* (1989) and is based on intrapancreatic injection of cells from the pancreatic carcinoma cell line (PC-1), which was originally derived from a BOP-induced cancer in Syrian golden hamsters. This method is relatively easy, requires less time for tumour induction and significantly limits the use of potentially hazardous carcinogens. It has been shown (Takiyama *et al.*, 1990) that this model shares many characteristics with human disease.

In two of the cited studies (Mang & Wieman, 1987; Schroeder *et al.*, 1988) Photofrin was used as the photosensitising agent, whilst Chatlani *et al.* (1992) studied aluminium sulphonated phthalocyanine (AlSPc). The alluring feature of 5-aminolaevulinic acid (ALA)-induced PPIX sensitisation, as used in this study, is the significantly decreased risk of prolonged skin photosensitivity. ALA can also be administered orally, as has been reported in human subjects, but only as a

**Figure 5** Kaplan-Meier survival curves for Syrian golden hamsters with transplanted pancreatic tumours comparing the PDT-treated group (■ = 9) with the control, non-treated group (●, $n = 4$) (log-rank test, $P < 0.02$).

single bolus dose (Berlin *et al.*, 1956; Grant *et al.*, 1993; Loh *et al.*, 1993*a*). We have compared two methods of oral ALA administration (bolus and fractionated). We anticipated that oral fractionated doses would result in better tissue uptake than an oral bolus because ALA and its metabolite, porphobilinogen, are very quickly excreted in the urine after a bolus dose (Berlin *et al.*, 1956). The peak plasma concentration of ALA in humans after a single dose is reached at 60 min and the half-life ($t_{1/2}$) of its clearance from the circulation is only about 50 min (Mustajoki *et al.*, 1992), so frequent repeated doses should maintain a steady, high blood level of ALA for a longer time. A similar approach was adopted in another recent study in which prolonged administration of ALA to rats over 11 days (in drinking water) resulted in a better liver tumour/normal liver PPIX ratio than shorter periods of administration (van Hillegersberg *et al.*, 1992). This tendency for better tumour uptake of ALA and accumulation of PPIX after fractionated as opposed to bolus dosing was confirmed in the present study. Another potential advantage of fractionating the ALA dose is that any asynchrony in PPIX synthesis among the malignant cells may be minimised. However, in this work the best results in terms of the efficiency of PPIX conversion from ALA were obtained after intravenous injection, as found previously in studies of normal rat stomach and colon (Loh *et al.*, 1993*a*). ALA arriving from the portal circulation after an oral dose can be quickly metabolised to porphyrins by hepatocytes *in situ* and then excreted into the bile and thus lost to the systemic circulation.

Recent studies of animal tissues after systemic ALA administration using chemical extraction have shown that the porphyrin content is at least 95% protoporphyrin IX (Loh *et al.*, 1993*b*). Good correlation was also found between chemically extracted levels of PPIX and microfluorimetric measurements using our technique in tissue layers of the gastrointestinal tract (Loh *et al.*, 1993*b*). In rat gastric and colonic mucosa, and in a duodenal tumour model, a fluorescence intensity of 100 counts per pixel (recorded with the 10 × objective to give the same calibrated sensitivity as the present work) was found to correspond to approximately 5–6 $\mu$g of PPIX per gram of tissue. Assuming that similar PPIX fluorescence efficiencies apply to the pancreatic tumour model, we would estimate PPIX levels of about 5 $\mu g\ g^{-1}$ 4 h after 200 $mg\ kg^{-1}$ i.v. administration. Despite the lack of absolute determination of PPIX levels, there are several advantages of the microfluorimetric technique compared with chemical extraction: firstly, we can monitor changes in the microscopic PPIX distribution which would not have been apparent from the gross extraction technique; and, secondly, using an H&E-stained serial section, it is easier with the microscopic technique to discriminate between viable and necrotic tumour. This is important, since PPIX is not synthesized in necrotic regions and with an extraction technique it is sometimes difficult to exclude sampling of necrotic areas. Normal pancreatic tissue showed relatively low fluorescence, regardless of the route of ALA administration, and much lower than other normal tissues (stomach, colon, bladder) studied by Loh *et al.* (1993*a*). The ratio between tumour and normal pancreatic tissue fluorescence levels in our study varied at different time points beween 1:1 and 8:1. For intravenous and oral (fractionated) administration the selectivity ratio between tumour and pancreas at the time of maximum tumour fluorescence was 5–6:1, although with oral (bolus) administration this ratio was lower at 3:1. However Mang and Wieman (1987) found no difference in Photofrin concentrations 24 h after injection between intrapancreatic tumours and normal pancreas in their rat and hamster models. In the study of Schroeder *et al.* (1988) the activity of $^{125}$I-labelled Photofrin was studied 3 and 48 h after administration and was almost twice as high in the pancreatic tumour as in the normal pancreas at both time points. In the case of aluminium sulphonated phthalocyanine (a mixture containing mono- to tetrasulphonates with the mean corresponding to trisulphonated) at the optimum time (48 h), the chemically extracted concentration (as measured using fluorescence detection) was about three times higher in the pancreatic tumours as in the normal pancreas (Chatlani *et al.*, 1992). Hence, using ALA-induced PPIX sensitisation the tumour to normal ratios demonstrate better selectivity than found with conventional exogenous sensitisation. Good selectivity has also been found in large bowel and experimental mammary carcinomas with this method of sensitisation (Bedwell *et al.*, 1992; Peng *et al.*, 1992). One of the possible explanations for this selective tumour accumulation of PPIX could be lower ferrochelatase activity in the tumour cells compared with normal tissues, as shown by van Hillegersberg (1992) and Schoenfeld *et al.* (1988) in combination with higher levels of porphobilinogen deaminase in tumour (Batlle, 1993). However, the selectivity found here is exceptional and must partly be explained by the relatively low PPIX fluorescence levels found in normal pancreas, which may also be a consequence of low activities of certain haem enzymes in this organ. On reaching maximum fluorescence levels in the pancreas, the PPIX appears to be associated with intercellular regions corresponding to the capillary and lymphatic network. A similar fluorescence pattern has been noted for the porphyrins and phthalocyanines (Nuutinen *et al.*, 1991). Further work with confocal imaging to give higher resolution would be desirable and might help to explain the resistance of normal pancreas to PDT damage, which appears to be a general phenomenon applying to all sensitisers studied thus far. When the same light dose was applied to the tumours and normal pancreas in the experiments of Mang and Wieman (1987) (tumour and normal pancreas had identical Photofrin concentrations), the damage was limited to tumour only. This phenomenon was accompanied by a notable lack of photobleaching of the Photofrin in normal tissues as compared with photobleaching in the tumour. It was suggested that normal pancreas contains high amounts of singlet oxygen scavengers which could maintain sublethal levels of singlet oxygen and also inhibit singlet oxygen-induced photodegradation. Chatlani *et al.* (1992) reported results with sulphonated aluminium phthalocyanine showing that the light dose necessary to induce necrosis in tumours resulted in no damage to normal pancreas. These authors also calculated that the threshold photodynamic dose for damage (light dose multiplied by concentration of photosensitiser) to normal pancreas was about seven times as high as for damage to pancreatic tumour. Studies trying to explain this phenomenon have been undertaken but are inconclusive (Matthew & Cui, 1990; Moesta *et al.*, 1992). Studies of photobleaching of ALA-induced PPIX and phthalocyanine in pancreas would provide a useful comparison with those performed with Photofrin. On the other hand, serious pancreatic damage was induced in another study (Schroder *et al.*, 1988) in which irradiation of the tumour was performed from a distance of about 3–4 cm, and most probably larger areas of normal pancreas and other organs around were irradiated.

This caused necrotising pancreatitis and death in one of seven animals. Another three animals also died from gastroduodenal perforations. The irradiation techniques described in the present study limited exposure to the pancreatic tumour and small areas of adjacent normal pancreas. Using this method only small areas of damage to the normal pancreas could be seen on histological examination, and these were at the places directly exposed to laser light. One cannot exclude the possibility that this was a result of thermal damage, which all PDT studies try to exclude. Many previous studies have reported small areas of damage where the fibre touches the target – so-called thermal controls (exposed to laser without previous sensitiser administration) (Barr *et al.*, 1987; Chatlani *et al.*, 1992).

The maximum depth of necrosis induced in the tumours by PDT in our study was 8 mm, with a viable rim of undamaged tumour tissue at the side opposite the irradiated area. Borders between necrotic areas and viable tumour appeared to follow the lobular structure of the tumour. This may be related to the vascular supply pattern of the tumour or perhaps the protective role of connective tissue strips dividing tumour into lobules. Of the three experimental pancreatic tumour PDT studies, only Chatlani *et al.* (1992) give a description of the localisation of the necrosis within the tumour. Their description is quite similar to our findings. They also reported a maximum of 8 mm necrosis of the tumour and a viable rim at the areas furthest from the site of light application. One can argue that the presence of a viable tumour rim should be considered an unsatisfactory result, but it is well established that light penetration is a limiting factor of PDT. If surface illumination, as used in our study, can produce damage up to 8 mm, this would be sufficient if PDT followed other tumour volume-reducing procedures such as surgery, and in these circumstances PDT would only aim at 'sterilising' resection margins. Other possibilities can also be considered, such as multiple fibres inserted at different depths into the tumour tissue to illuminate larger volumes.

No other studies have presented survival analysis after PDT for pancreatic tumours. This study shows favourable results, with significant prolongation of survival after treatment. However, all animals had a tumour recurrence, most probably from the rims of viable tumour observed in our short-term study or from incompletely destroyed microscopic foci of tumour.

In summary, the results of this study suggest that pancreatic tumours up to 8 mm in depth can be destroyed following ALA administration and application of 50 J $cm^{-2}$ of 630 nm laser light. Partial destruction of the tumour results in a prolongation of survival of experimental animals. These data would justify further studies of PDT for pancreatic cancer, which should also consider how pancreatic tumours could be treated in clinical practice. Imaging of pancreatic lesions is difficult and leads to delays in diagnosis and treatment. Therefore the most probable application of PDT in this disease would be as an adjunct to surgery. In this situation destruction of tumour to a depth of up to 8 mm, as found here, can be sufficient to destroy micrometastases in the operative area, and for small tumours PDT could be used as the primary treatment. Intraoperative PDT is already being seriously considered (DeLaney *et al.*, 1993; Evrad *et al.*, 1993) and hopefully will become established in the future treatment of intra-abdominal malignancy.

This project was funded by the Association for International Cancer Research. Additionally, we should like to thank Deprenyl USA (for support for Miss J. Bedwell), the British Council (for supplementary support for Dr J. Regula), Mrs A Burt for assistance with tissue culture and Dr G. Buonaccorsi for help with the laser. Professor S.G. Bown is funded by the Imperial Cancer Research Fund.

### References

BARR, H., TRALAU, C.J., MACROBERT, A.J., KRASNER, N., BOULOS, P.B., CLARK, C.G. & BOWN, S.G. (1987). Photodynamic therapy in the normal rat colon with phthalocyanine sensitisation. *Br. J. Cancer*, **56**, 111–118.

BATTLE, DEL C.A.M. (1993). Porphyrins, porphyrias, cancer and photodynamic therapy–a model for carcinogenesis. *J. Photochem. Photobiol. B: Biol.*, **20**, 5–22.

BEDWELL, J., MACROBERT, A.J., PHILLIPS, D. & BOWN, S.G. (1992). Fluorescence distribution and photodynamic effect of ALA-induced PPIX in the DMH rat colonic tumour model. *Br. J. Cancer*, **65**, 818–824.

BERLIN, N.I., NEUBERGER, A. & SCOTT, J.J. (1956). The metabolism of δ-aminolaevulinic acid. 1. Normal pathways, studied with the aid of $^{15}N$. *Biochem. J.*, **64**, 80–90.

BOWN, S.G. (1990). Photodynamic therapy to scientists and clinicians – one world or two? *J. Photochem. Photobiol. B: Biol.*, **6**, 1–12.

CHATLANI, P.T., NUUTINEN, P.J.O., TODA, N., BARR, H., MACROBERT, A.J., BEDWELL, J. & BOWN, S.G. (1992). Selective necrosis in hamster pancreatic tumours using photodynamic therapy with phthalocyanine photosensitisation. *Br. J. Surg.*, **79**, 786–790.

DELANEY, T.F., SINDELAR, W.F., TOCHNER, Z., SMITH, P.D., FRIAUF, W.S., THOMAS, G., DACHOWSKI, L., COLE, J.W., STEINBERG, S.M. & GLATSTEIN, E. (1993). Phase I study of debulking surgery and photodynamic therapy for disseminated intraperitonal tumours. *Int. J. Radiat. Oncol.*, **25**, 445–457.

DIVARIS, D.X.G., KENNEDY, J.C. & POTTIER, R.H. (1990). Phototoxic damage to sebaceous glands and hair follicles of mice after systemic administration of 5-aminolevulinic acid correlates with localized protoporphyrin IX fluorescence. *Am. J. Pathol.*, **136**, 891–897.

DOUGHERTY, T.J., COOPER, M.T. & MANG, T.S. (1990). Cutaneous phototoxic occurrences in patients receiving Photofrin. *Lasers Surg. Med.*, **10**, 485–488.

EGAMI, H., TAKIYAMA, Y., CANO, M., HOUSER, W.H. & POUR, P.M. (1989). Establishment of hamster pancreatic ductal carcinoma cell line (PC-1) producing blood group-related antigens. *Carcinogenesis*, **10**, 861–869.

EVRAD, S., APRAHAMIAN, M. & MARESCAUX, J. (1993). Intra-abdominal photodynamic therapy: from theory to feasibility. *Br. J. Surg.*, **80**, 298–303.

GRANT, W.E., HOPPER, C., MACROBERT, A.J., SPEIGHT, P.M. & BOWN, S.G. (1993). Photodynamic therapy of oral cancer: photosensitisation with systemic aminolaevulinic acid. *Lancet*, **342**, 147–148.

KENNEDY, J.C. & POTTIER, R.H. (1992). Endogenous protoporphyrin IX, a clinically useful photosensitiser for photodynamic therapy. *J. Photochem. Photobiol., B: Biol.*, **14**, 275–292.

LOH, C.S., MACROBERT, A.J., BEDWELL, J., REGULA, J., KRASNER, N. & BOWN, S.G. (1993*a*). Oral versus intravenous administration of 5-aminolaevulinic acid for photodynamic therapy. *Br. J. Cancer*, **68**, 41–51.

LOH, C.S., VERNON, D.I., MACROBERT, A.J., BEDWELL, J., BOWN, S.G. & BROWN, S.B. (1993*b*). Endogenous porphyrin distribution induced by 5 aminolaevulinic acid in the tissue layers of the gastrointestinal tract. *J. Photochem. Photobiol. B: Biol.*, **20**, 47–54.

MALIK, Z. & LUGACI, H. (1987). Destruction of erythroleukaemic cells by photoactivation by endogenous porphyrins. *Br. J. Cancer*, **56**, 589–595.

MANG, T.S. & WIEMAN, T.J. (1987). Photodynamic therapy in the treatment of pancreatic carcinoma: dihematoporphyrin ether uptake and photobleaching kinetics. *Photochem. Photobiol.*, **46**, 853–858.

MATHEWS, E.K. & CUI, Z.J. (1990). Photodynamic action of sulphonated aluminium phthalocyanine (SALPC) on AR4-2J cell, a carcinoma cell line of rat exocrine pancreas. *Br. J. Cancer*, **61**, 695–701.

MOESTA, K.T., HURLEY, E.L. & MANG, T.S. (1992). Intercellular organization determines the differential PDT-sensitivities of two human pancreatic cancer lines in vitro. In *Photodynamic Therapy and Biomedical Lasers*, Spinelli, P., Dal Fante, M. & Marchesini, R. (eds) pp. 292–296. Excerpta Medica: Amsterdam.

MUSTAJOKI, P., TIMONEN, K., GORCHEIN, A., SEPPALAINEN, A., MATIKAINEN, E. & TENHUNEN, R. (1992). Sustained high plasma 5-aminolaevulinic acid concentration in a volunteer: no porphyric symptoms. *Eur. J. Clin. Invest.*, **22**, 407-411.

NUUTINEN, P.J.O., CHATLANI, P.T., BEDWELL, J., MACROBERT, A.J., PHILLIPS, D. & BOWN, S.G. (1991). Distribution and photodynamic effect of disulphonated aluminium phthalocyanine in the pancreas and adjacent tissues in the Syrian golden hamster. *Br. J. Cancer*, **64**, 1108-1115.

PENG, Q., MOAN, J., WARLOE, T., NESLAND, J.M. & RIMINGTON, C. (1992). Distribution and photosensitising efficiency of porphyrins induced by application of exogenous 5-aminolaevulinic acid in mice bearing mammary carcinoma. *Int. J. Cancer*, **52**, 433-443.

SCHOENFELD, N., EPSTEIN, O., LAHAV, M., MAMET, R., SHAKLAI, M. & ATSMON, A. (1988). The haem biosynthetic pathway in lymphocytes of patients with malignant lymphoproliferative disorders. *Cancer Lett.*, **43**, 43-48.

SCHRODER, T., CHEN, I.-W., SPERLING, M., BELL, R.H., BRACKETT, K. & JOFFE, S.N. (1988). Hematoprophyrin derivative uptake and photodynamic therapy in pancreatic carcinoma. *J. Surg. Oncol.*, **38**, 4-9.

TAKIYAMA, Y., EGAMI, H. & POUR, P.M. (1990). Expression of human tumour-associated antigens in pancreatic cancer induced in Syrian hamsters. *Am. J. Pathol.*, **136**, 707-715.

TRALAU, C.J., YOUNG, A.R., WALKER, N.P.J., VERNON, D.I., MACROBERT, A.J., BROWN, S.B. & BOWN, S.G. (1989). Mouse skin photosensitivity with dihaematoporphyrin ether (DHE) and sulphonated phthalocyanine (A1SPc): a comparative study. *Photochem. Photobiol.*, **49**, 305-312.

VAN HILLEGERSBERG, R., VAN DEN BERG, J.W.O., KORT, W.J., TERPSTRA, O.T. & WILSON, J.H.P. (1992). Selective accumulation of endogenously produced porphyrins in a liver metastasis model in rats. *Gastroenterology*, **103**, 647-651.


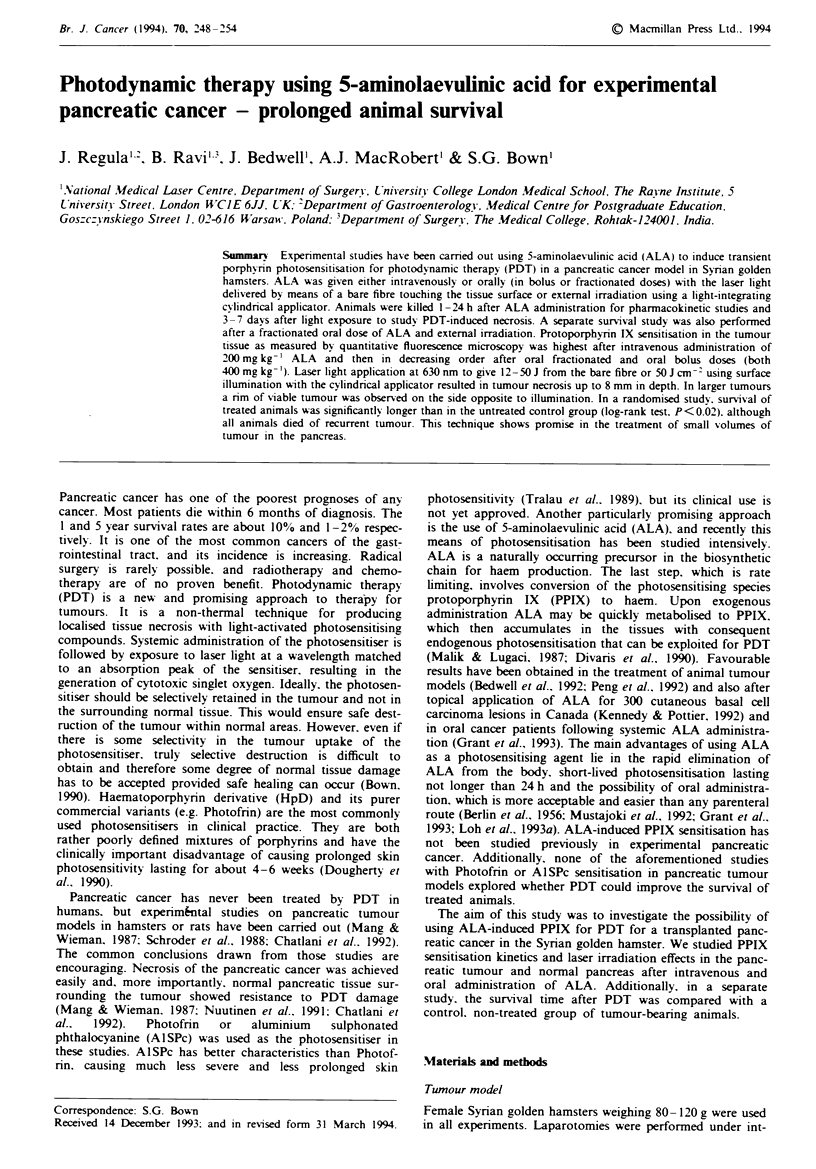

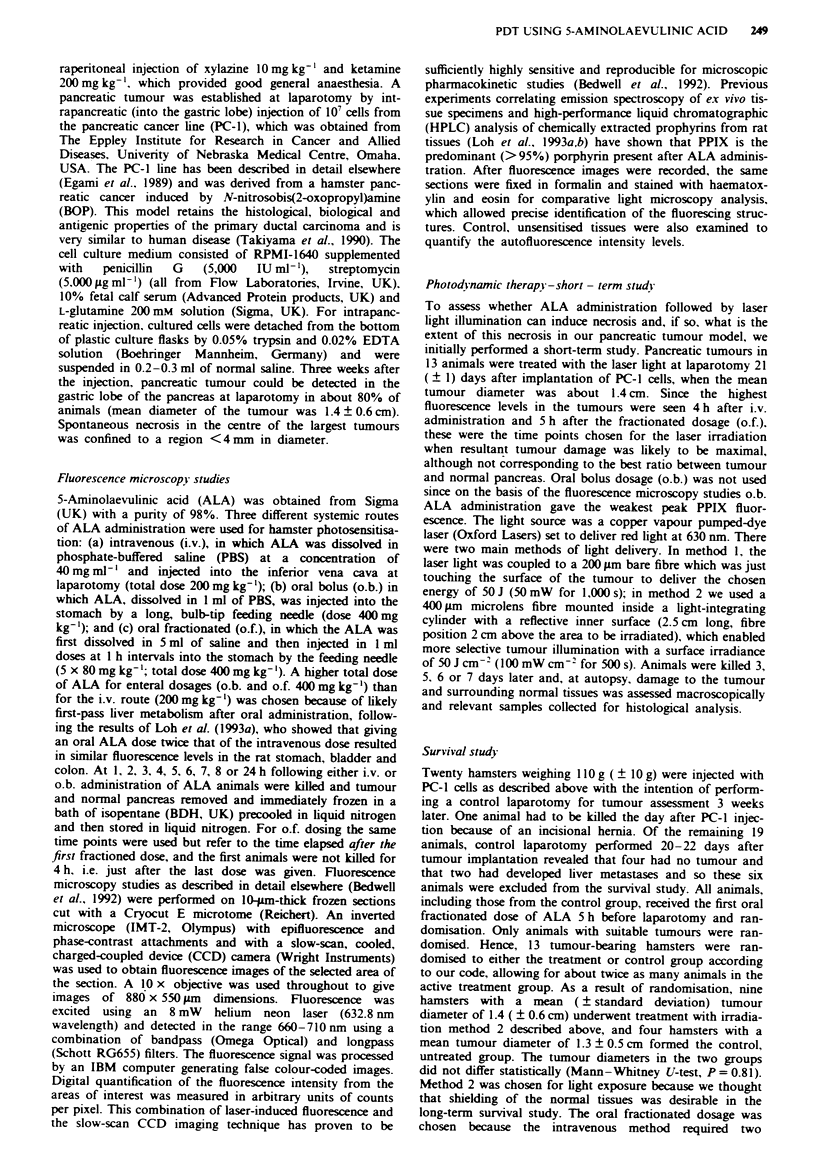

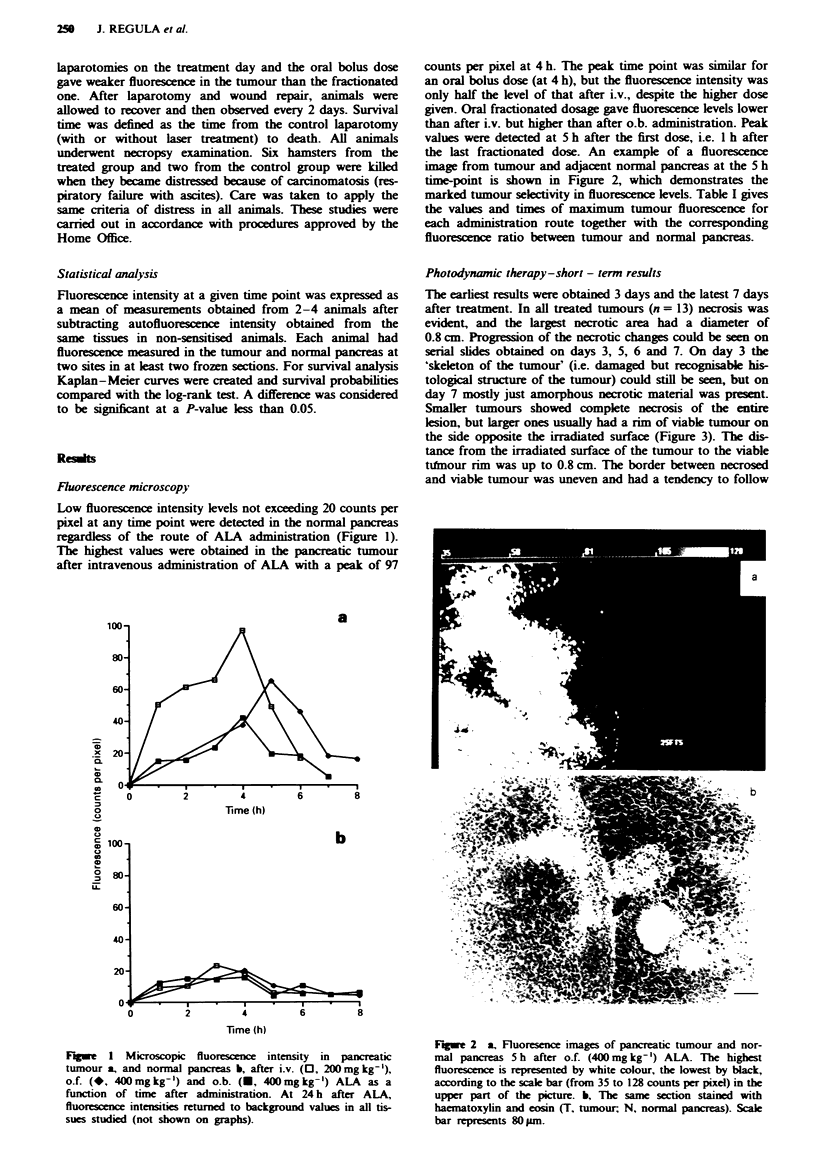

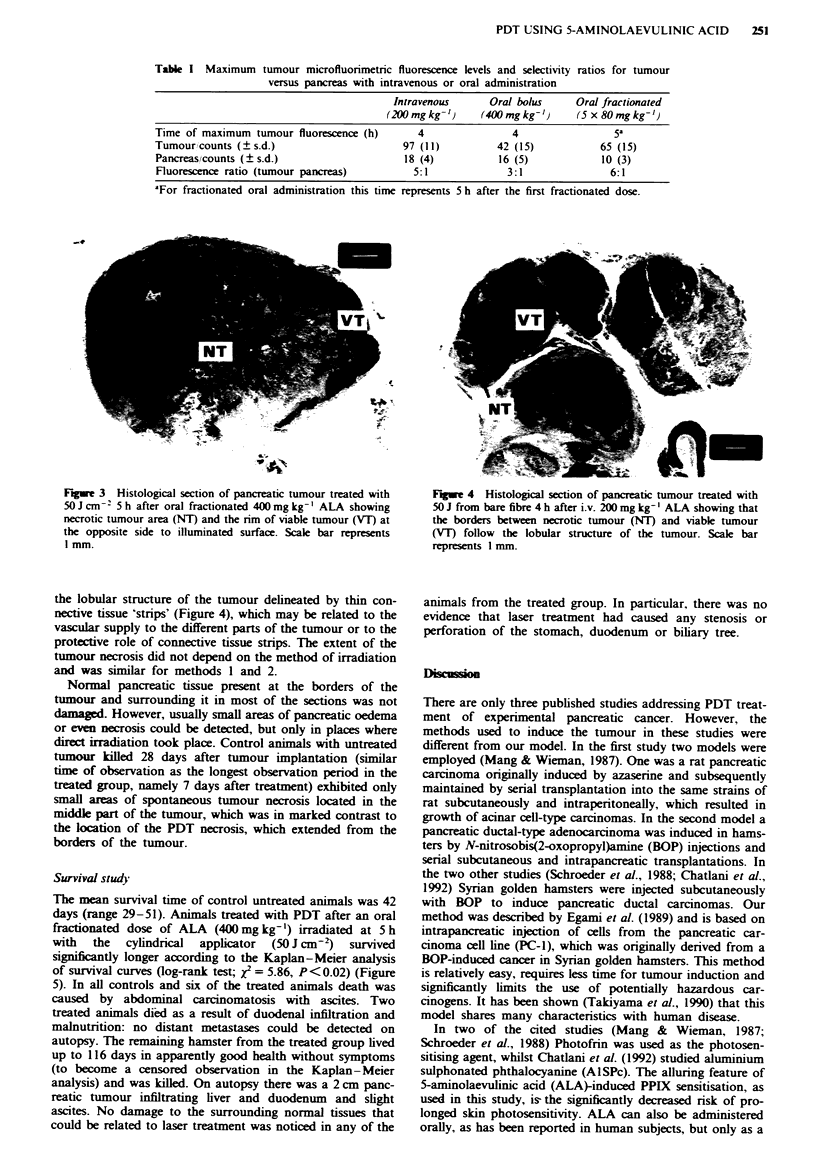

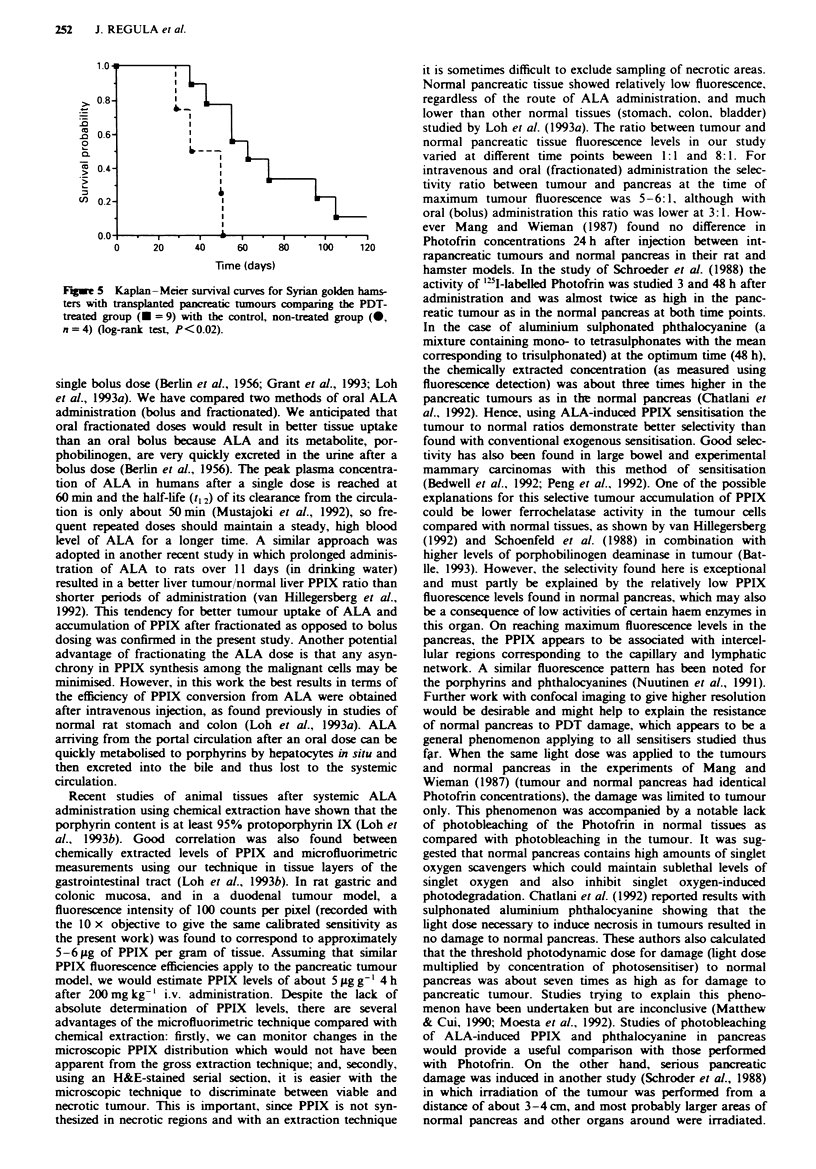

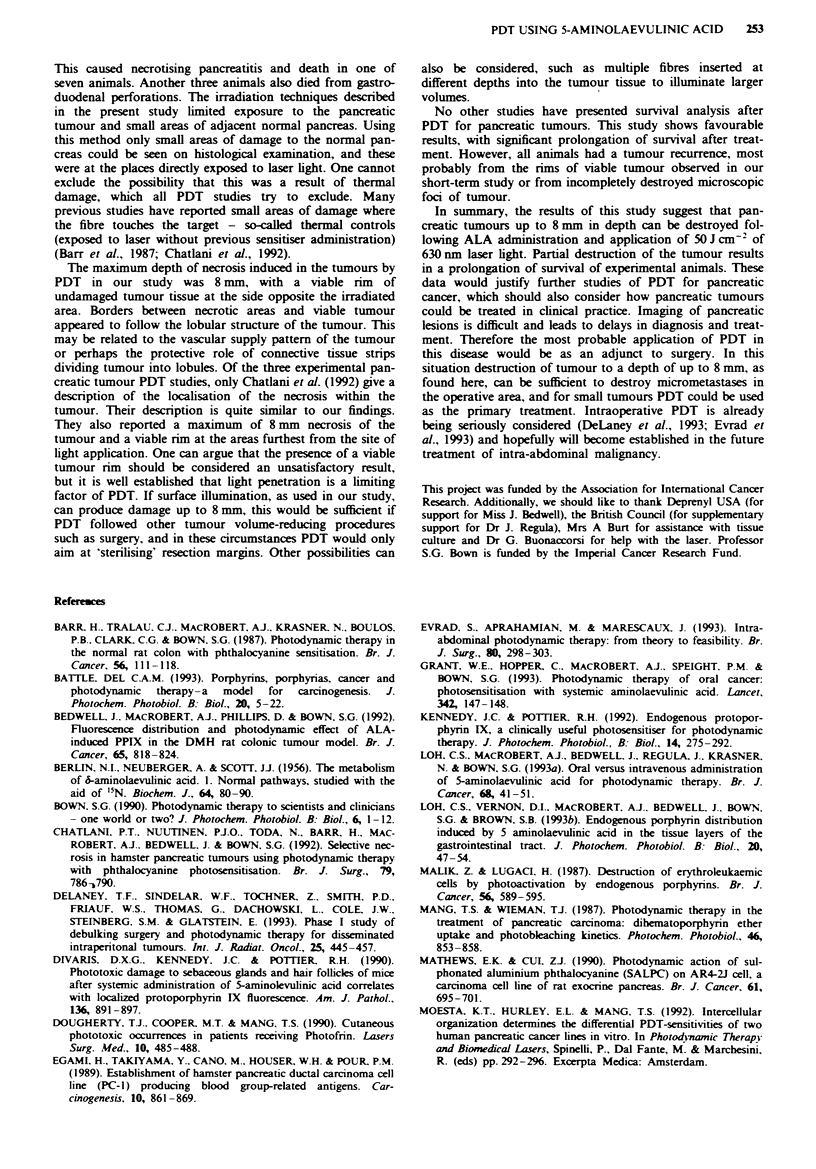

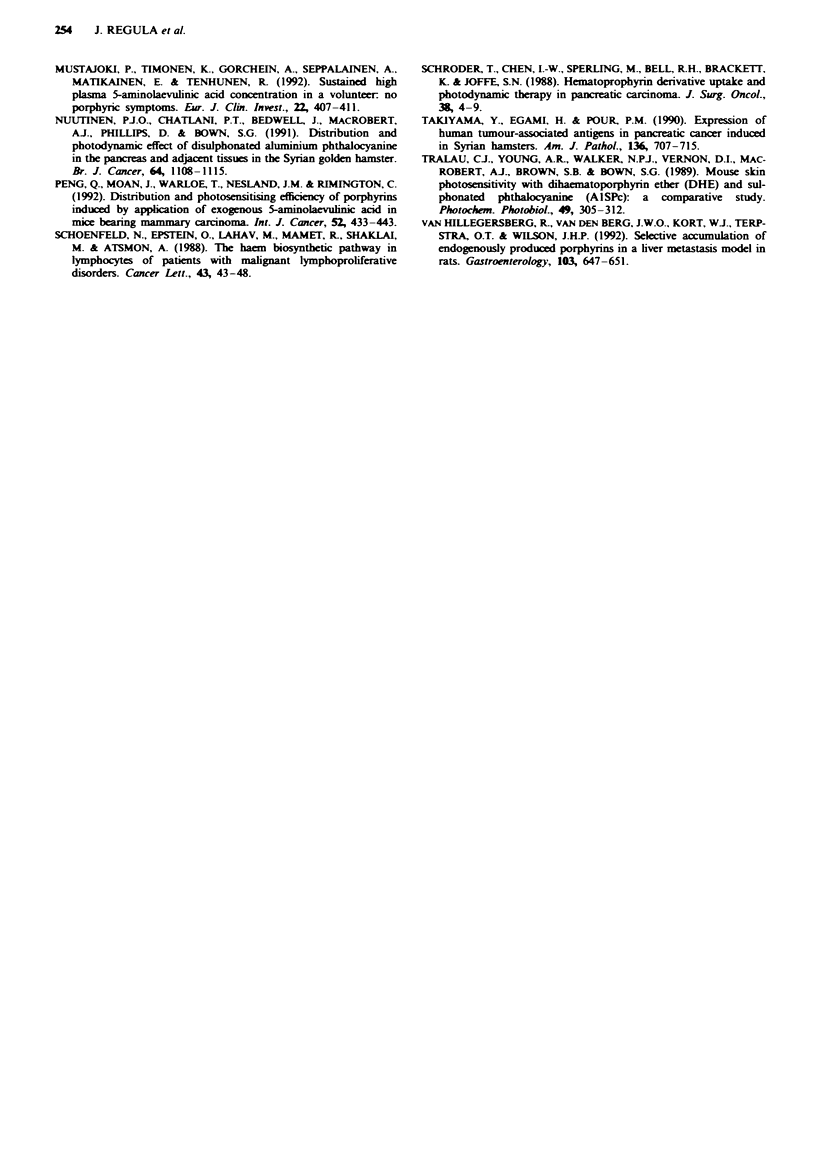


## References

[OCR_00767] BERLIN N. I., NEUBERGER A., SCOTT J. J. (1956). The metabolism of delta -aminolaevulic acid. 1. Normal pathways, studied with the aid of 15N.. Biochem J.

[OCR_00753] Barr H., Tralau C. J., MacRobert A. J., Krasner N., Boulos P. B., Clark C. G., Bown S. G. (1987). Photodynamic therapy in the normal rat colon with phthalocyanine sensitisation.. Br J Cancer.

[OCR_00761] Bedwell J., MacRobert A. J., Phillips D., Bown S. G. (1992). Fluorescence distribution and photodynamic effect of ALA-induced PP IX in the DMH rat colonic tumour model.. Br J Cancer.

[OCR_00772] Bown S. G. (1990). Photodynamic therapy to scientists and clinicians--one world or two?. J Photochem Photobiol B.

[OCR_00775] Chatlani P. T., Nuutinen P. J., Toda N., Barr H., MacRobert A. J., Bedwell J., Bown S. G. (1992). Selective necrosis in hamster pancreatic tumours using photodynamic therapy with phthalocyanine photosensitization.. Br J Surg.

[OCR_00782] DeLaney T. F., Sindelar W. F., Tochner Z., Smith P. D., Friauf W. S., Thomas G., Dachowski L., Cole J. W., Steinberg S. M., Glatstein E. (1993). Phase I study of debulking surgery and photodynamic therapy for disseminated intraperitoneal tumors.. Int J Radiat Oncol Biol Phys.

[OCR_00789] Divaris D. X., Kennedy J. C., Pottier R. H. (1990). Phototoxic damage to sebaceous glands and hair follicles of mice after systemic administration of 5-aminolevulinic acid correlates with localized protoporphyrin IX fluorescence.. Am J Pathol.

[OCR_00796] Dougherty T. J., Cooper M. T., Mang T. S. (1990). Cutaneous phototoxic occurrences in patients receiving Photofrin.. Lasers Surg Med.

[OCR_00801] Egami H., Takiyama Y., Cano M., Houser W. H., Pour P. M. (1989). Establishment of hamster pancreatic ductal carcinoma cell line (PC-1) producing blood group-related antigens.. Carcinogenesis.

[OCR_00807] Evrard S., Aprahamian M., Marescaux J. (1993). Intra-abdominal photodynamic therapy: from theory to feasibility.. Br J Surg.

[OCR_00814] Grant W. E., Hopper C., MacRobert A. J., Speight P. M., Bown S. G. (1993). Photodynamic therapy of oral cancer: photosensitisation with systemic aminolaevulinic acid.. Lancet.

[OCR_00820] Kennedy J. C., Pottier R. H. (1992). Endogenous protoporphyrin IX, a clinically useful photosensitizer for photodynamic therapy.. J Photochem Photobiol B.

[OCR_00823] Loh C. S., MacRobert A. J., Bedwell J., Regula J., Krasner N., Bown S. G. (1993). Oral versus intravenous administration of 5-aminolaevulinic acid for photodynamic therapy.. Br J Cancer.

[OCR_00829] Loh C. S., Vernon D., MacRobert A. J., Bedwell J., Bown S. G., Brown S. B. (1993). Endogenous porphyrin distribution induced by 5-aminolaevulinic acid in the tissue layers of the gastrointestinal tract.. J Photochem Photobiol B.

[OCR_00836] Malik Z., Lugaci H. (1987). Destruction of erythroleukaemic cells by photoactivation of endogenous porphyrins.. Br J Cancer.

[OCR_00843] Mang T. S., Wieman T. J. (1987). Photodynamic therapy in the treatment of pancreatic carcinoma: dihematoporphyrin ether uptake and photobleaching kinetics.. Photochem Photobiol.

[OCR_00847] Matthews E. K., Cui Z. J. (1990). Photodynamic action of sulphonated aluminium phthalocyanine (SALPC) on AR4-2J cells, a carcinoma cell line of rat exocrine pancreas.. Br J Cancer.

[OCR_00862] Mustajoki P., Timonen K., Gorchein A., Seppäläinen A. M., Matikainen E., Tenhunen R. (1992). Sustained high plasma 5-aminolaevulinic acid concentration in a volunteer: no porphyric symptoms.. Eur J Clin Invest.

[OCR_00868] Nuutinen P. J., Chatlani P. T., Bedwell J., MacRobert A. J., Phillips D., Bown S. G. (1991). Distribution and photodynamic effect of disulphonated aluminium phthalocyanine in the pancreas and adjacent tissues in the Syrian golden hamster.. Br J Cancer.

[OCR_00875] Peng Q., Moan J., Warloe T., Nesland J. M., Rimington C. (1992). Distribution and photosensitizing efficiency of porphyrins induced by application of exogenous 5-aminolevulinic acid in mice bearing mammary carcinoma.. Int J Cancer.

[OCR_00880] Schoenfeld N., Epstein O., Lahav M., Mamet R., Shaklai M., Atsmon A. (1988). The heme biosynthetic pathway in lymphocytes of patients with malignant lymphoproliferative disorders.. Cancer Lett.

[OCR_00892] Takiyama Y., Egami H., Pour P. M. (1990). Expression of human tumor-associated antigens in pancreatic cancer induced in Syrian hamsters.. Am J Pathol.

[OCR_00897] Tralau C. J., Young A. R., Walker N. P., Vernon D. I., MacRobert A. J., Brown S. B., Bown S. G. (1989). Mouse skin photosensitivity with dihaematoporphyrin ether (DHE) and aluminium sulphonated phthalocyanine (AlSPc): a comparative study.. Photochem Photobiol.

[OCR_00906] Van Hillegersberg R., Van den Berg J. W., Kort W. J., Terpstra O. T., Wilson J. H. (1992). Selective accumulation of endogenously produced porphyrins in a liver metastasis model in rats.. Gastroenterology.

